# How Informative is the Immune Response Against Surrogate Tumor Antigens to Assess Antitumor Immunity?

**DOI:** 10.3389/fonc.2014.00135

**Published:** 2014-06-04

**Authors:** Valérie Janelle, Alain Lamarre

**Affiliations:** ^1^Immunovirology Laboratory, Institut National de la Recherche Scientifique, INRS-Institut Armand-Frappier, Laval, QC, Canada; ^2^Department of Biology, Biomed Research Center, Université du Québec à Montréal, Montréal, QC, Canada

**Keywords:** oncolytic viruses, tumor antigens, CD8-positive T-lymphocytes, B16F10, VSV

The last decade has seen the development of numerous antitumor therapeutic approaches. Concomitantly, the interest for using oncolytic viruses (OV) against cancer has grown tremendously and a number of promising candidates are now in preclinical and clinical studies. Tumor regression *in vivo* following viral infection has been shown to be a multifactorial process (1). The reductionist view of viruses simply causing direct lysis of infected cancer cells has now been replaced by a view including the complex interplay between viruses and the tumor environment. The important role of the immune response in either limiting or enhancing OV therapy is also now well recognized (2, 3). The prototypic *Rhabdoviridae* VSV has generated encouraging results in various experimental tumor models and is now used in a phase I clinical trial in patients with liver cancer (www.clinicaltrials.gov; #NCT01628640). VSV possesses intrinsic oncolytic properties as it replicates more efficiently in type-I interferon (IFN)-defective cells, a pathway frequently impaired during tumorigenesis (4). Cancer therapy using VSV has been shown to generate a variety of immune responses including tumor-specific CD8^+^ T cells that are induced following the release of TA by infected cells (5). However, the tumor-specific immune response generated following VSV treatment is usually weak and often only leads to transient tumor control. Experimental tumor models expressing various surrogate non-self-TA have been developed over the years to more easily assess the magnitude and quality of immune responses generated against tumors. However, whether these responses are always representative of physiological antitumor immune responses is unclear.

Recently, our group characterized various VSV glycoprotein (G) mutants capable of interfering with host cell metabolism by inhibiting cellular transcription and translation in a kinetic similar to WT VSV as opposed to the prototypic matrix (M) mutant (M_M51R_) that is slightly attenuated *in vitro* (6). Furthermore, VSV G mutants proved to be more cytolytic for B16 melanoma cells *in vitro* than the M mutant. To analyze their oncolytic potential *in vivo*, we used an immunocompetent mouse model implanted with B16 tumors transfected with a DNA minigene encoding the immunodominant CD8^+^ T cell epitope of the lymphocytic choriomeningitis virus (glycoprotein aa 33–41) (7) as a surrogate non-self-TA (B16gp33) (8). Mice were injected subcutaneously into the flank with B16gp33 cells and when tumors reached a palpable size (day 7), animals were treated intratumorally every second day with three doses (days 7, 9, and 11) of WT VSV or of the G or M mutants. Tetramer and intracellular cytokine staining analysis revealed that CD8^+^ T cells harvested from mice treated with WT VSV or the G mutants developed a polyfunctional gp33-specific immune response. Surprisingly however, the strength of the gp33-specific immune response generated did not correlate with the ability of a particular strain of VSV to slow down parental B16 growth and improve mice survival. Treatment with WT VSV was the poorest at controlling B16 tumor progression even though it induced a strong CTL response against gp33. On the other hand, M_M51R_ was more efficient than WT VSV at slowing down B16 growth despite the fact that this virus induced the lowest gp33-specific T cell response. We therefore determined whether CD8^+^ T cell responses directed against endogenous self-TA were involved in limiting tumor progression. CTL responses against self-TA, such as TRP-1 and gp100, were barely detectable *ex vivo* when analyzed separately. However, adoptive transfer of purified CD8^+^ T cells harvested from M_M51R_-treated B16gp33 melanoma-bearing mice into naive mice provided better protection against parental B16 tumor implantation compared to CTLs taken from WT or G mutant-treated mice. These results suggest that the M mutant, despite being the weakest at inducing a T cell response against the surrogate non-self-TA gp33, induces the broadest antitumoral CTL response.

B16 melanoma is a highly aggressive tumor model in part because major histocompatibility complex class I (MHC-I) surface expression is very low on these cells. Strikingly, B16 infection with VSV M mutant induced the upregulation of surface MHC-I both *in vitro* and *in vivo*, a phenomenon that was not observed for WT VSV of the G mutants (8). The matrix protein of VSV was previously shown to alter trafficking of a molecule structurally similar to MHC-I, namely CD1d (9, 10). This leads to inhibition of antigen presentation to natural killer T (NKT) cells (11). Thus, VSV matrix protein could participate in the retention of MHC-I molecules within infected cells while the mutated protein in M_M51R_ may lack this ability. Thus, surface MHC-I upregulation following M_M51R_ treatment likely explains the significantly improved CD8^+^ T cell-dependent survival despite the poor gp33-specific CTL response induced by this mutant. This may subsequently lead to presentation of a broader pool of B16 TA proportionally reducing the response against gp33 (see Figure 1 for model).

**Figure 1:**
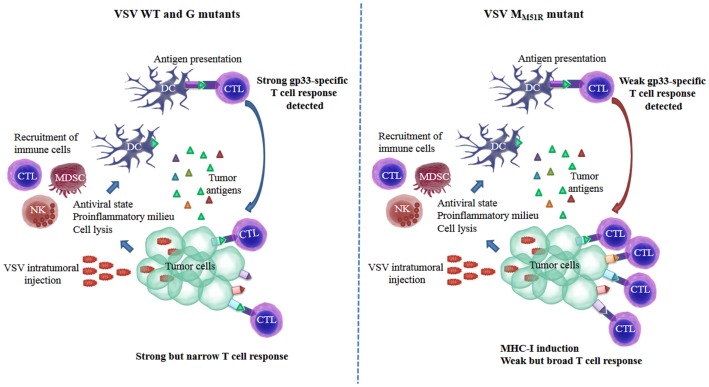
**Induction of a functional tumor-specific immune response is achieved through diverse mechanisms for different VSV strains: proposed model**. VSV is quickly cleared from tumor tissue by the rapid induction of innate antiviral defense mechanisms and neutralizing antibodies. Nonetheless, the proinflammatory milieu generated in response to infection promotes leukocyte infiltration. Infection can result in tumor cell lysis either directly as a result of virus replication or indirectly through the action of innate immune cells generating a pool of tumor-associated antigens that may be taken-up by antigen-presenting cells such as dendritic cells and lead to T lymphocyte activation. Infection with WT VSV or glycoprotein (G) mutants induces a strong CD8^+^ cytotoxic T lymphocyte (CTL) response against a surrogate non-self antigen (gp33) correlating with their ability to limit tumor growth (left panel). In contrast, the matrix mutant of VSV (M_M51R_), although inefficient at inducing gp33-specific CTLs, is highly effective at slowing down tumor progression, likely through its capacity to induce the upregulation of MHC-I surface expression on cancer cells allowing for the induction of a broader CTL response (right panel). CTLs, cytotoxic T lymphocytes; DC, dendritic cells; MDSC, myeloid-derived suppressor cell; MHC-I, major histocompatibility complex class I; NK, natural killer; VSV, vesicular stomatitis virus.

In a recent study, Pedersen et al. compared vaccine-induced CD8^+^ T cell responses directed against self and non-self-TA and showed that vaccination with adenoviral vectors encoding endogenous TA had little or no effect on the growth of B16 melanomas whereas vaccination with a similar vector construct expressing a surrogate non-self-TA induced efficient tumor control (12). Although vaccination against both self and non-self-TA induced comparable CD8^+^ T cell responses in terms of cell numbers and effector functions, CTLs directed against self-TA were of lower functional avidity. These results are in agreement with our study and provide a potential mechanism explaining why T cell responses against self and non-self-TA are different and might not be induced at proportional levels during OV therapy.

Taken together, these results highlight a considerable limitation of many experimental systems used to assess antitumor immunity and warrant caution when extrapolating responses against surrogate TA to the overall antitumoral immune response. This may prove critical for the development of novel or improved OV, which may be biased by incorrectly estimating immune response correlates using such experimental systems. Therefore, great efforts will need to be made to develop improved methods for analyzing the antitumoral immune response induced by OVs against a broader array of TA in order to better appreciate their full therapeutic potential.

## Conflict of Interest Statement

The authors declare that the research was conducted in the absence of any commercial or financial relationships that could be construed as a potential conflict of interest.
